# Effect of random terms on the shape of dendrites analyzed by the phase-field model

**DOI:** 10.1371/journal.pone.0324281

**Published:** 2025-08-04

**Authors:** Shinya Fujiwara, Kiyoshiro Okada, Katsuhiro Endo, Kenta Hirayama, Mayu Muramatsu

**Affiliations:** 1 Department of Science for Open and Environmental Systems, Graduate School of Keio University, Yokohama, Kanagawa, Japan; 2 Research Center for Computational Design of Advanced Functional Materials, National Institute of Advanced Industrial Science and Technology (AIST), Tsukuba, Ibaraki, Japan; University of Vigo, SPAIN

## Abstract

In this study, we focus on random terms with a significant effect on the calculation time and the sidebranch structure of dendrites. We quantitatively evaluate the effect of the random terms on dendrites by changing the term introduced into the evaluation equation, the distribution of the random term, and variance. We introduced the terms: χ and ∇·q. In the phase-field equation, we use χ to represent interfacial noise. In the heat conduction equation, we use ∇·q to represent thermal noise. We compare the results of calculation using only χ with those using only ∇·q and analyze the effect of each random term introduced on the shape of dendrites. In terms of the probability distribution for generating random numbers, the uniform and Gaussian distributions are used for comparison. The magnitude of the noise *F*_*u*_, which controls the variance of noise, is used with 3 or 4 patterns of values to the extent that the calculation results do not diverge. Each value of *F*_*u*_ differs by a factor of 10. Sidebranch length, contour length, and area are used as indices for evaluating dendrites. The succinonitril is used for comparing the simulation and experimental results for dendrites to design the optimal random number model. On the basis of the introduced terms, we clarify that each term requires a different order of the variance. The probability density function of random numbers does not affect the shape of dendrites. On the other hand, the calculation time which is used the random numbers following the uniform distribution is more than twice as fast as when using random numbers that follow a Gaussian distribution. The variance of a random term has the greatest effect on the shape of dendrites.

## 1 Introduction

### 1.1 Phase-field model for dendrite growth

Materials are often formed in intricately branched crystals in solid form. They are called dendrites, commonly seen in an undercooled melt [1,2]. Predicting the growth of a dendrite is extremely important for improving material properties because the dendrite is a common structure formed in the solidification of many commercial metals, and the shape of dendrites has significant impacts on metal properties [3–5]. Some studies have shown the metallic material solidification by synchrotron X-ray imaging [4,6–8]. Also, the dendritic architecture changes material surface characteristics. The growth induction of dendritic architecture has also attracted attention. Ivantsov’s solution provides a foundational analytical framework for modeling steady-state dendrite growth by solving the heat diffusion equation for an isothermal paraboloid in a uniformly supercooled melt [9]. This solution can be applied to both two-dimensional (cylindrical) and three-dimensional (spherical) geometries, enabling analytical descriptions of dendritic growth in various spatial dimensions. This approach effectively predicts the relationship between undercooling and dendrite tip velocity but lacks a mechanism for selecting the unique tip radius. This limitation was later addressed by the microscopic solvability theory, which introduced the concept that anisotropic surface tension stabilizes dendritic growth and determines the tip radius [10]. While these classical models have significantly contributed to our understanding of dendritic solidification, they rely on simplified assumptions, such as steady-state growth, and do not naturally accommodate the effects of solute diffusion, convection, or complex microstructural interactions. In contrast, the phase-field method provides a more comprehensive framework for simulating dendritic growth, as it can inherently capture the evolution of complex interface morphologies without explicitly tracking the solid-liquid boundary [11]. Unlike Ivantsov’s analytical solution, which assumes a fixed tip shape, or the microscopic solvability theory, which assumes infinitesimally small perturbations, the phase-field method enables dynamic tracking of morphological instabilities and solute interactions in both binary and multi-component systems [12]. Hsu *et al*. found the relationship between external driving force and character patterns of dendritic architecture by using the phase-field model [13]. Therefore, there has been great discussion about simulating the growth of dendrites, and some numerical techniques for dendrite growth have been established [14–17]. In particular, the phase-field method is the most widely used for simulating dendrite growth [13,18–22].

**Fig 1:**
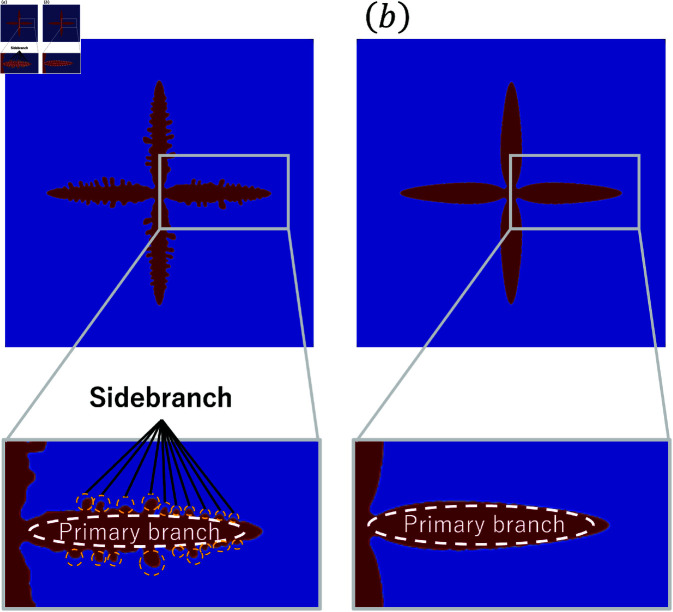
Results of the phase-field analysis (a) with (b) without using random term.

As shown in Fig 1(a), dendrites can be evaluated on the basis of their two structures. The first is the primary branch, which has a tree-trunk-like structure. The second is the sidebranches, which are derived from the primary branch. The sidebranches are used for analyzing dendrites [23,24]. In the phase-field method, random numbers are used to represent sidebranches. In this study, we use the sidebranches to evaluate the morphological characteristics of dendrites, and we analyze the relationship between the random terms and calculation times.

### 1.2 Random terms in the phase-field model

The phase-field method is characterized by the adoption of an order parameter referred to as the phase-field variable ϕ. The phase-field method enables us to calculate the time variation by using driving and diffusion terms. We utilize the Allen-Cahn equation, which is used for nonconserved value, to minimize the total energy of the system. Furthermore, a heat conduction equation incorporating the latent heat generation of dendrites was formulated based on the conservative law of energy. The basic equations of this model are expressed below.

$$\frac{\partial \phi }{\partial t}=-M_{\phi }\left(\frac{\partial F}{\partial \phi }+\chi \right)$$ (1)

$$\frac{\partial T}{\partial t}=\kappa \nabla^{2}T+\frac{L}{c}\frac{\partial p(\phi )}{\partial \phi }\frac{\partial \phi }{\partial t}-\nabla \cdot q$$ (2)

Here, χ and q are the random terms referring to the fluctuations of driving force and thermal noise, respectively. Additionally, $M_{\phi }$ is the phase-field mobility coefficient, *F* is the Gibbs free energy function, *T* is the temperature, κ is the thermal diffusion coefficient, *L* is the latent heat, and *c* is the specific heat. Eq (1) is the time evolution equation of the phase-field variable, and χ is generally utilized in the phase-field method [25,26]. Karma and Rappel [27] used ∇·q instead of χ as a random term in the heat conduction equation shown in Eq (2). The random term determines complex shapes in dendrite growth. Fig 1 shows the results of the calculation. Fig 1(a) shows the dendrite obtained by using the random term and Fig 1(b) shows that obtained without using the random term. It indicates that the random term determines the shape of dendrites in terms of branched crystal structures, such as the primary branch and sidebranches. Random terms have an essential role in the development of sidebranches. Kobayashi [25] used a random term that follows a uniform distribution for χ to represent the instability of the shape of the interfaces against the noise. This noise is phenomenologically set and used to reproduce the interface dynamics. In Kobayashi’s model, thermodynamic noise based on the Langevin equation is not considered, and therefore ∇·q is not used. On the other hand, Karma and Rappel [27] used a random term that follows the Gaussian distribution for ∇·q. There have been only a few studies of the types of random term and the distribution of random number, in which the variance of random terms is determined empirically. Little attention has been given to the accuracy of the amplitude of the variance.

**Fig 2:**
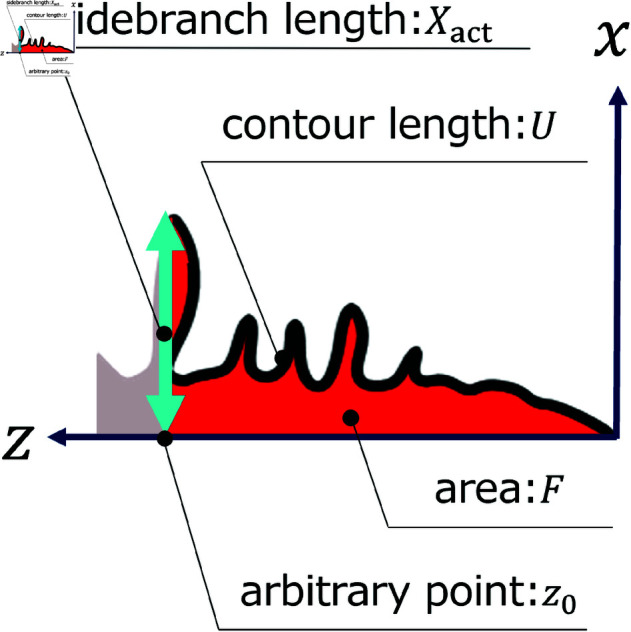
Schematic of the coordinate system and the dendrite indices.

In this research study, we focus on the type, distribution, and variance of random terms in the phase-field method. The material simulated in this study is succinonitrile (SCN), whose dendrites have been extensively evaluated [28–32]. The relationship between sidebranch length and the distance between the tip of the primary branch and a arbitrary point *z*_0_ on the *z* axis in the Fig 2 is indicated by the power law. In addition, not only sidebranch length but also contour length and area to the distance between the tip of the primary branch and the arbitrary point *z*_0_ are indicated by the power law, as shown in previous studies [33–36]. Moreover, we calculate the coefficient of power law and compare them in the basis of random terms, distribution, and variance. We compare these coefficients across different random term conditions and experimental results in terms of morphological characteristics. Next, we compare computation times to identify a more suitable random number model, as each random number distribution employs a different generation process. We then discuss the suitable model for the random term in the phase-field model. To simplify this problem, we employ the sharp interface model [37], the most fundamental form of the phase-field method.

## 2 Methods

### 2.1 Phase-field method

#### 2.1.1 Fundamental phase-field model.

**Fig 3:**
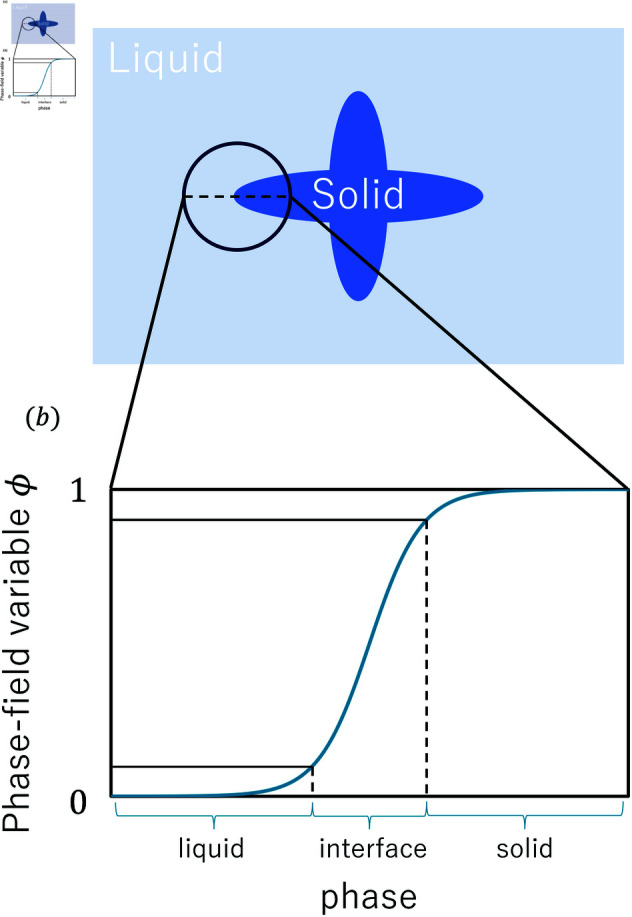
Representation of the phase in the phase-field variable (a) image of the dendrite and (b) correspondence between the phase-field variable and the solid–liquid interface phase.

The phase-field method is widely used to simulate the processes associated with meso-scale material compositions, such as interface migration [38], phase transformation [39] and grain growth [40]. Various studies have established the phase-field method to simulate dendritic growth in pure materials [25,41,42]. The phase-field method introduces the order parameter ϕ. The phase-field ϕ varies gradually in the solid–liquid interface region as shown in Fig 3. In this simulation, ϕ is solid when ϕ=1 and liquid when ϕ=0. Owing to ϕ, complicated boundary-following calculations are not necessary. The phase-field method has the advantage of enabling the easy simulation of dendrite growth. The order parameter ϕ in the dendrite growth model represents the phase state. The time evolution of dendrite growth is expressed as

$$\frac{\partial \phi }{\partial t}=-M_{\phi }\frac{\delta }{\delta \phi }\int_{V}\left(f_{grad}+f_{chem}+f_{doub}\right)dv,$$ (3)

where $f_{grad}$ is the gradient energy density, $f_{chem}$ is the chemical free energy density, and $f_{doub}$ is the double-well potential. These are defined as follows.

$$f_{grad}=\frac{\varepsilon^{2}}{2}|\nabla \phi {|}^{2}$$ (4)

$$f_{chem}=p(\phi )f_{S}+(1-p(\phi ))f_{L}\;=f_{L}+p(\phi )\left(f_{S}-f_{L}\right)$$ (5)

$$f_{doub}=Wq(\phi )$$ (6)

Here, ε is the gradient coefficient, p(ϕ) is the monotonically increasing function, *f*_*S*_ and *f*_*L*_ are the bulk free energies in the solid and liquid, respectively, *W* is the height of the energy barrier, and q(ϕ) is a double-well function. In this case, p(ϕ) and q(ϕ) are expressed as

$${\begin{array}{c} p(\phi )=\phi^{3}(10-15\phi +6\phi^{2})\\ q(\phi )=\phi^{2}{\left(1-\phi \right)}^{2} \end{array}\}}_{.}$$ (7)

The gradient coefficient ε is expressed as

$$\varepsilon (\theta )=\overset{―}{\varepsilon }\left[1+\zeta \cos\left\{k\left(\theta -\theta_{0}\right)\right\}\right],$$ (8)

where $\overset{―}{\varepsilon }$ is the average of ε, ζ is the strength of anisotropy, *k* is the anisotropy mode, θ is the angle between the *x*-axis and the normal vector of the interface, and $\theta_{0}$ is the angle between the *x*-axis and the vector of primary arm growth. Considering Eqs (3)–(6), we obtain the equation.

$$F=\int_{V}\left\{\frac{\varepsilon (\theta {)}^{2}}{2}|\nabla \phi {|}^{2}+f_{bulk}(\phi )\right\}dv$$ (9)

Here, the free bulk energy $f_{bulk}$ is expressed as $f_{bulk}(\phi )=f_{chem}+f_{doub}$. The functional derivative $\frac{\delta F}{\delta \phi }$ can be given by Eq (9).

$$\frac{\delta F}{\delta \phi }=-\nabla \cdot \left(\varepsilon (\theta {)}^{2}\nabla \phi -\varepsilon (\theta )\frac{\partial \varepsilon (\theta )}{\partial \theta }\nabla^{2}\phi \right)+\frac{\partial f_{bulk}(\phi )}{\partial \phi }$$ (10)

Substituting Eqs (5)–(7) into *f*_*doub*_ of Eq (10), we can obtain the following equation.

$$\frac{\delta F}{\delta \phi }=-\nabla \cdot \left(\varepsilon (\theta {)}^{2}\nabla \phi -\varepsilon (\theta )\frac{\partial \varepsilon (\theta )}{\partial \theta }|\nabla \phi {|}^{2}\right)\;-4W\phi (1-\phi )\left\{\phi -\frac{1}{2}-\frac{15\Delta f}{2W}\phi (1-\phi )+\chi \right\}$$ (11)

Here, Δf is the difference in energy between the solid and liquid phases, and χ is the random term. Furthermore, Δf is expressed as

$$\Delta f=f_{S}-f_{L}=\frac{L\left(T-T_{m}\right)}{T_{m}}.$$ (12)

Eq (12) shows that Δf consists of the latent heat *L*, the temperature *T* and the melting point *T*_*m*_. Substituting Eqs (10) and (12) into Eq (3), we can obtain the following phase-field equation.

$$\frac{\partial \phi }{\partial t}=M_{\phi }[\nabla \cdot \left(\varepsilon (\theta {)}^{2}\nabla \phi -\varepsilon (\theta )\frac{\partial \varepsilon (\theta )}{\partial \theta }|\nabla \phi {|}^{2}\right)+\;.4W\phi (1-\phi )\left\{\phi -\frac{1}{2}-\frac{15}{2W}\frac{L\left(T-T_{m}\right)}{T_{m}}\phi (1-\phi )+\chi \right\}]$$ (13)

In the phase-field method of simulating solidification, the parameters are as follows.

$$\overset{―}{\varepsilon }=\sqrt{\frac{3\delta \gamma }{b}},\;W=\frac{6\gamma b}{\delta }$$ (14)

$$M_{\phi }=\frac{T_{m}\mu \sqrt{2W}}{6\overset{―}{\varepsilon }L}=\frac{bT_{m}\mu }{3\delta L}$$ (15)

Here, μ is the interfacial kinetic coefficient, γ is the interfacial energy, δ is the interface thickness, *b* is the contant defined as $b=2\tanh^{-1}(1\;-\;2\lambda )$, and λ is the interfacial area determination coefficient. Through the application of the sharp interface model [37], the mobility $M_{\phi }$ is defined and scaled to be consistent with the interface velocity. In the sharp interface model, the interface thickness δ is assumed to converge to 0. Then, the phase-field mobility coefficient $M_{\phi }$ is defined to be consistent with the Stephan condition, which defines the interface velocity, and the Gibbs-Thomson effect, which accounts for the influence of interface curvature [43,44].

The heat conduction equation should be solved when we use a pure material in the phase-field method, as shown in Eq (2). The second term on the right-hand side of Eq (2) is the generation term introduced to generate latent heat during the change from the liquid phase to the solid phase. Substituting Eq (7) into Eq (2), we obtain the following heat conduction equation.

$$\frac{\partial T}{\partial t}=\kappa \nabla^{2}T+30\frac{L}{c}\phi^{2}(1-\phi {)}^{2}\frac{\partial \phi }{\partial t}-\nabla \cdot q$$ (16)

In order to determine the initial temperature $T_{init}$, we introduce dimentionless undercooling Δ. The relation is the following equation:

$$T_{init}=T_{m}-\frac{L}{c}\Delta$$ (17)

#### 2.1.2 Random terms in the phase-field model.

Karma and Rappel have shown the variances of χ and ∇·q [27].

$$\left\langle \chi_{ij}\chi_{i^{\prime }j^{\prime }}\right\rangle =\frac{2\Lambda JF_{u}}{\Delta t\Delta x^{2}}\delta_{ii^{\prime }}\delta_{jj^{\prime }}$$ (18)

$$\left\langle q_{m,ij}q_{n,i^{\prime }j^{\prime }}\right\rangle =\frac{2\kappa F_{u}}{\Delta t\Delta x^{2}}\delta_{mn}\delta_{ii^{\prime }}\delta_{jj^{\prime }}$$ (19)

Here, $\delta_{ij}$ is Kronecker’s delta, Λ is the inverse of capillary length, *J* is the numerical constant, *F*_*u*_ is the magnitude of noise, Δt is the time step, and Δx is the lattice of spacing. Various *F*_*u*_ values are used to evaluate the effect on its dendrite shape.

**Fig 4:**
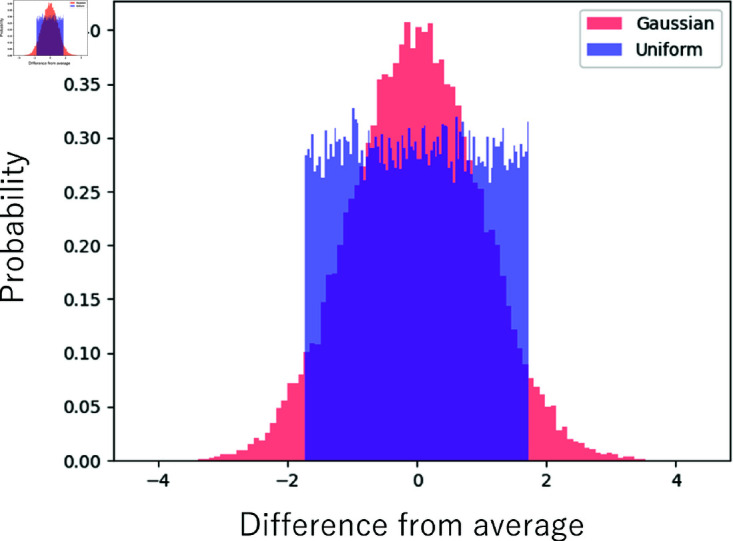
Uniform distribution and Gaussian distribution of random number used in this study.

In this study, Mersenne Twister [45] is employed as the random number generator. Mersennne Twister generates a sequence of pseudo-random numbers on the basis of an incremental formula by giving true random numbers as a random number seed. In terms of distribution, Merssenne Twister generates random numbers following a uniform distribution. Random numbers following a Gaussian distribution are generated by Box–Muller transformation [46]. Transformation converts random numbers following a uniform distribution into random numbers following a Gaussian distribution. Fig 4 shows an example of the uniform and Gaussian distributions utilized in this study for generating random numbers.

### 2.2 Measurement of the characteristics of dendrite

**Fig 5:**
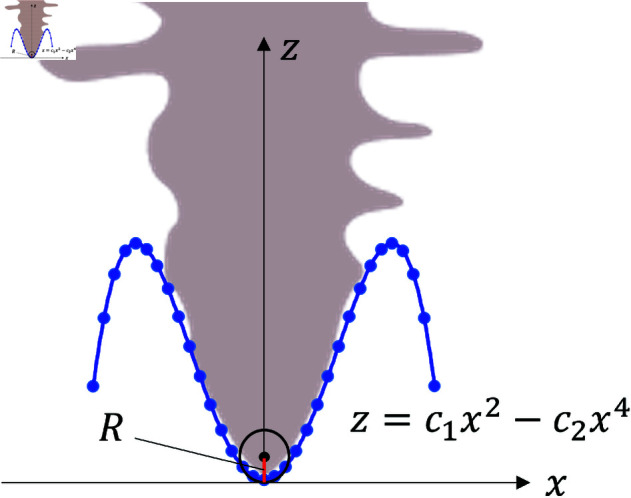
Scaling of tip radius.

The dendrites are evaluated using three indices, i.e., the sidebranch length *X*_*act*_, the contour length *U*, and the area *F*. These indices are normalized by the tip radius to enable the quantitative comparison of dendrite shapes. First, the dendrite tip shape is approximated as

$$z=c_{1}x^{2}-c_{2}x^{4},$$ (20)

where *z* is the distance between the tip of the primary branch and the arbitrary point on the *z* axis and *x* is the distance from the central axis to the surface of the dendrite (Fig 5). Eq (20) represents a refined version of Ivantsov’s solution, incorporating the effects of anisotropy and surface tension [47]. The coefficients *c*_1_ and *c*_2_ are derived by applying this equation to the tip shape depending on the difference in random number. The relationship between the tip radius *R* and the coefficients is expressed as follows.

$$R=\frac{1}{2c_{1}}$$ (21)

The fitting range must be considered in deriving the tip radius. We define the tip as the range from the top of primary branch to the first sidebranch that appears. The tip contour is approximated by Eq (20). As mentioned above, three evaluation indices are compared: the sidebranch length $X_{act}$, the contours length *U*, and the area *F* in Fig 2. The sidebranch length $X_{act}$ is defined as the length from the center axis of the primary branch to the tip of the sidebranch. The indices $X_{act}$, *U*, *F*, *z*, and *R* can be approximated by the power law [33–36]. The following equations provide the approximate formulas for each indicator.

$$\frac{X_{act}}{R}=a_{X}{\left(\frac{z}{R}\right)}^{b_{X}}$$ (22)

$$\frac{U}{R}=a_{U}{\left(\frac{z}{R}\right)}^{b_{U}}$$ (23)

$$\frac{F}{R^{2}}=a_{F}{\left(\frac{z}{R}\right)}^{b_{F}}$$ (24)

The coefficients *a*_*i*_ and *b*_*i*_(*i* = *X*,*U*,*F*) are evaluated by comparing the experimental data with the results of all of these analyses with different random numbers. Table 1 shows the experimental values. Table 2 shows the computational parameters and properties of SCN. In this study, several values of the anisotropy strength ζ are analyzed. We simulate dendrites by the phase-field method and evaluate $a_{X},a_{U},a_{F},b_{X},b_{U},$ and *b*_*F*_. In this study, we simulate dendrites 20 times in different random seeds for smoothing. We use Intel Xeon Gold 6136 for calculation.

**Table 1:** Coefficients of the power law in SCN dendrite [33,34,36].

sidebranch length $X_{act}$	$a_{X}=0.67,b_{X}=0.86$
contour length *U*	$a_{U}=0.38,b_{U}=1.50$
area *F*	$a_{F}=0.58,b_{F}=1.72$

**Table 2:** Phase-field computational parameters and properties of SCN.

lattice	1200×1200 [27]
lattice spacing Δx	0.8 μm [48]
anisotropy mode *k*	4 [49]
interface energy γ	$8.9\times 10^{-3}\;J/m^{2}$ [49–51]
interfacial kinetic coefficient μ	$2.05\times 10^{-2}\;m/\left(K\cdot s\right)$ [49]
thermal conductivity *K*	0.223 J/(m·K·s) [50,51]
specific heat *c*	$1.99\times 10^{6}\;J/\left(m^{3}\cdot K\right)$ [49,51]
latent heat *L*	$4.78\times 10^{7}\;J/m^{3}$ [49,51]
melting point *T*_*m*_	331.23 K [50,51]
time step	100000 [27]
dimensionless undercooling Δ	0.2
time step Δt	$7.14\times 10^{-10}\;s$
anisotropy strength ζ	[0.015,0.020,0.025,0.030]

## 3 Results and discussion

### 3.1 Comparison of results obtained using χ and ∇·q

**Fig 6:**
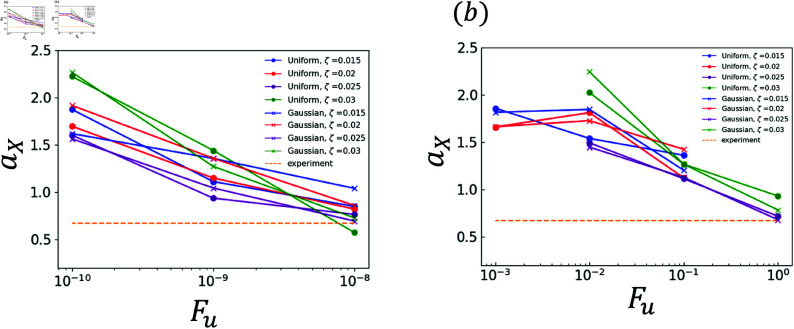
Coefficient *a*_*X*_ for *F*_*u*_ in Eq (22) with respect to the sidebranch length *X*_*act*_ (a) Effect of difference in χ (b) Effect of difference in ∇·q.

**Fig 7:**
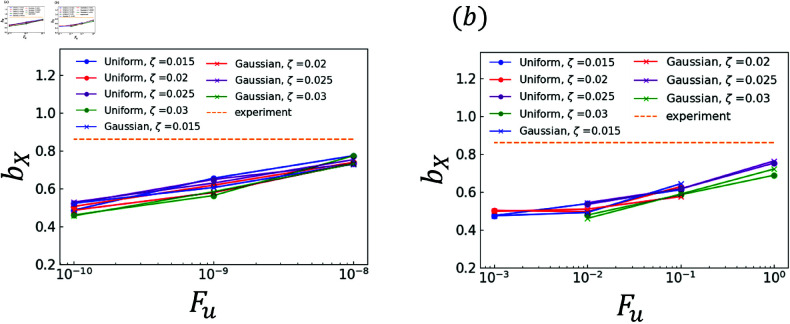
Coefficient *b*_*X*_ for *F*_*u*_ in Eq (22) with respect to the sidebranch length *X*_*act*_ (a) Effect of difference in χ (b) Effect of difference in ∇·q.

**Fig 8:**
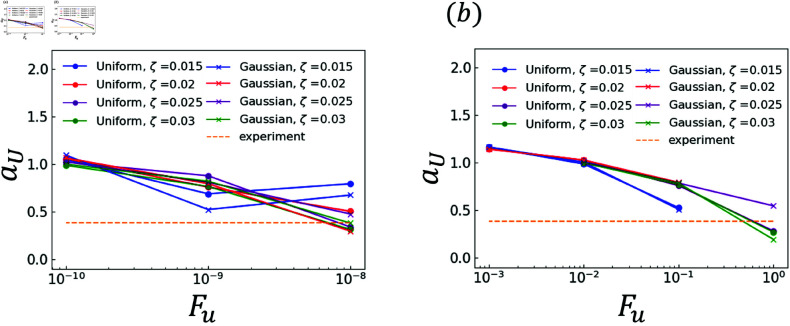
Coefficient *a*_*U*_ for *F*_*u*_ in Eq (23) with respect to the contour length U (a) Effect of difference in χ (b) Effect of difference in ∇·q.

**Fig 9:**
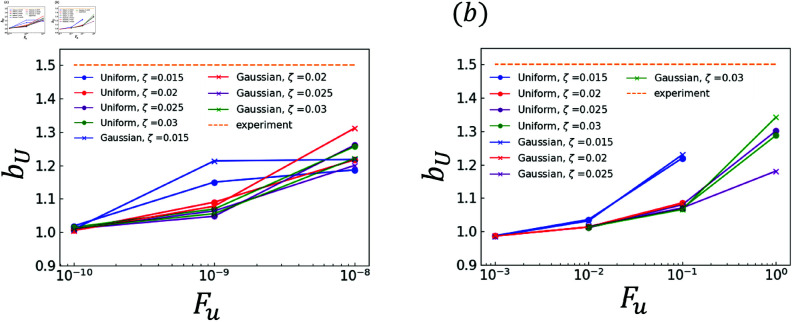
Coefficient *b*_*U*_ for *F*_*u*_ in Eq (23) with respect to the contour length U (a) Effect of difference in χ (b) Effect of difference in ∇·q.

**Fig 10:**
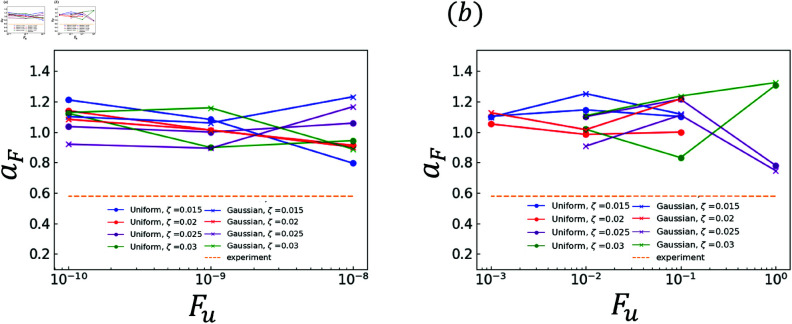
Coefficient *a*_*F*_ for *F*_*u*_ in Eq (24) with respect to the area F (a) Effect of difference in χ (b) Effect of difference in ∇·q.

**Fig 11:**
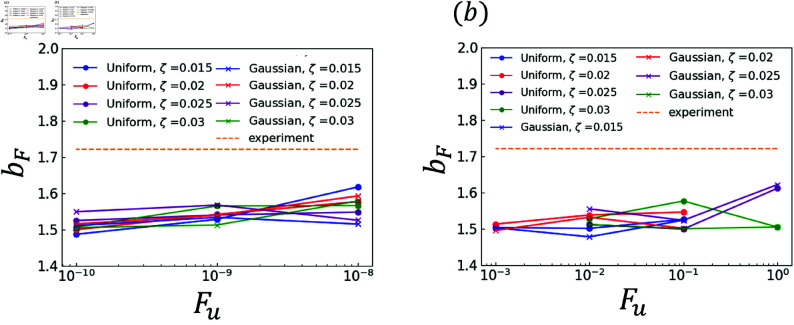
Coefficient *b*_*F*_ for *F*_*u*_ in Eq (24) with respect to the area F (a) Effect of difference in χ (b) Effect of difference in ∇·q.

The effects of random terms are investigated. Specifically, the analysis results obtained using χ and those obtained using ∇·q are compared. The experimental and analytical results are plotted in Figs 6–11. Dendrites are not generated correctly at several *F*_*u*_ value. The parameters for *F*_*u*_ that form dendrites correctly are shown in Figs 6–11. Fig 6 shows the results of the coefficient *a*_*X*_ for the sidebranch length *X*_*act*_. Fig 7 shows the results of the coefficient *a*_*X*_ for the sidebranch length *X*_*act*_. In Figs 6 and 7, the ranges of *a*_*X*_ and *b*_*X*_ are almost the same between the results obtained using χ and those obtained using ∇·q. On the other hand, the range of *F*_*u*_ is different. When χ is used, *F*_*u*_ takes a value from 10^−10^ to 10^−8^. When ∇·q is used, *F*_*u*_ takes a value from 10^−3^ to 10^−0^. The main reason for the difference is the difference in range between ΛJ and κ in Eqs (18) and (19). Figs 8–11 show the same trend as Figs 6 and 7. We can observe the difference in *F*_*u*_ range between the results obtained using χ and ∇·q

Another reason for the difference in *F*_*u*_ range in which normal dendrites are observed is that the phase-field variable ϕ ranges from 0 to 1, so the analysis introducing χ requires the variance of the random numbers in Eq (18) to be sufficiently small so as not to exceed the ϕ range. On the other hand, the analysis introducing ∇·q depends on the melting point. The range taken by the temperature field *T* is wider than ϕ, so the variance of the random term ∇·q becomes larger than that of the random term χ.

### 3.2 Comparison of distributions

As indicated by Figs 6–11, almost all of the circles and x marks show the same value. The value with random numbers following the uniform distribution almost agrees with that with random numbers following the Gaussian distribution in the results of the sidebranch length, contour length, and area. This finding is attributed to the central limit theorem. When random numbers act, the sum of random numbers approaches the Gaussian distribution, even if they follow a uniform distribution. Consequently, the difference between distributions cannot be confirmed.

According to Karma’s study [52], owing to the Langevin formalism, random numbers are governed by the Gaussian distribution in the theoretical formula. However, this result indicates that a uniform distribution can also be used in the numerical analysis by the phase-field method.

### 3.3 Comparison between numerical and experimental results

In Figs 6–9, the simulated sidebranch and contour lengths are closer to the experimental values with the increase in *F*_*u*_. When χ is used for the phase-field method, its better value for *F*_*u*_ is $1.0\times 10^{-8}$. When ∇·q is used for the phase-field method, its better value for *F*_*u*_ is 1.0. As the magnitude of the noise *F*_*u*_ increases, the variance of the random term increases, which allows a sidebranch to develop. Therefore, the simulated sidebranch length *X*_*act*_ and contour length *U* are expected to approach the experimental values with increasing *F*_*u*_. In addition, since *a*_*F*_ and *b*_*F*_, which are the coefficients of the area *F*, do not change when *F*_*u*_ and ζ are changed as shown in Figs 10 and 11, it is confirmed that the random term does not affect the area *F*. The reason is associated with the sum of random numbers. The average of the random number term converges to zero. Fig 13 shows the analysis results for different *F*_*u*_ values, and it can be seen that the sidebranch grows as *F*_*u*_ increases. When *F*_*u*_ is small, the sidebranch can hardly be seen, but the primary branch becomes thicker. When *F*_*u*_ increases, the sidebranch grows, but the primary branch becomes thinner. This is the reason why no changes can be observed in the coefficients of the area.

**Fig 12:**
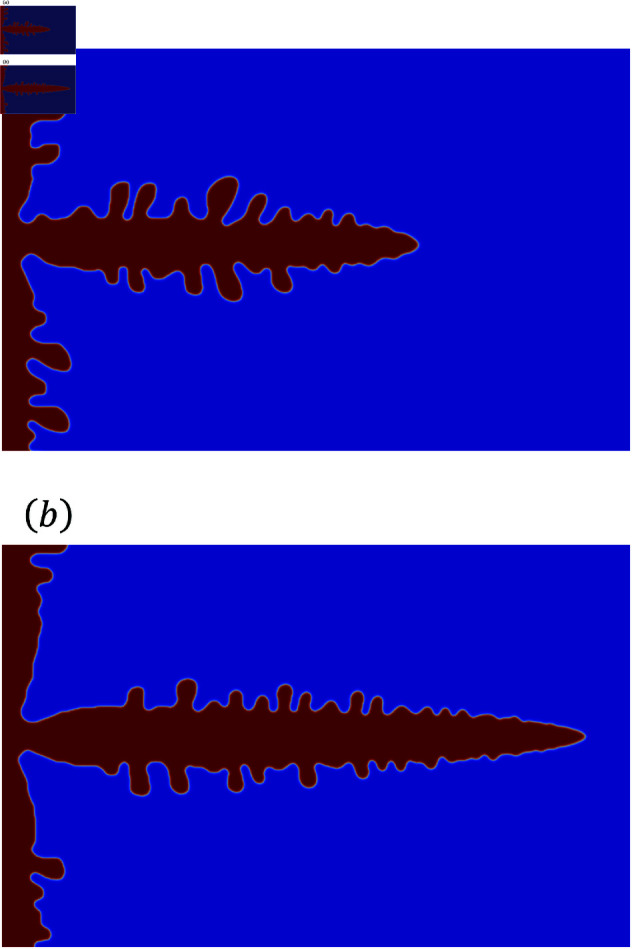
Comparison the shape of dendrites under different anisotropy strength. (a) anistropy strength ζ is 0.015. (b) anistropy strength ζ is 0.025.

**Fig 13:**
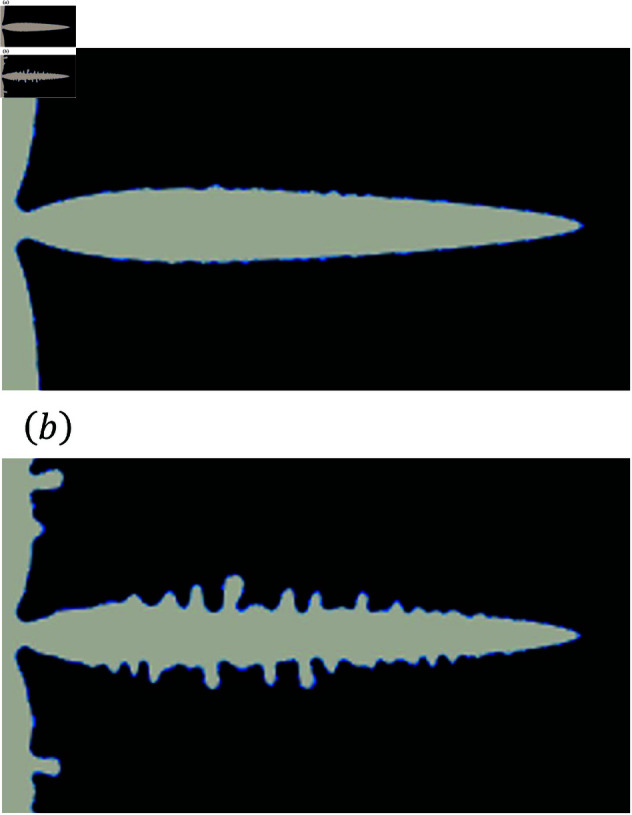
Comparison of analysis results for different *F*_*u*_ values at the anisotropy strength ζ=0.02. (a) The magnitude of the noise *F*_*u*_ is $1.0\times 10^{-10}$. (b) The magnitude of the noise *F*_*u*_ is $1.0\times 10^{-8}$.

On the other hand, when the anisotropy strength is low, the dendrite shape is highly influenced by random terms and cannot accommodate random numbers with large variance. As shown in Fig 12, the growth along the primary branch axis is more pronounced with higher anisotropy strength. Therefore, the growth in the direction of sidebranches is suppressed, resulting in a stable shape even when random numbers with large variance are applied. However, with low anisotropy strength, the growth becomes more sensitive to random numbers, as it tends to occur in directions perpendicular to the primary branch axis. Consequently, when random numbers with large variance are applied, the influence of random terms becomes excessive, preventing the formation of dendrite-like structures. Moreover, significant differences in the coefficients $a_{X},a_{U},a_{F},b_{X},b_{U}$, and *b*_*F*_ for each anisotropy strength are not clearly observed. This is because the sidebranch length decreases,which results in the tip radius becoming smaller as the anisotropy strength increases, as shown in Fig 12. These findings are consistent with previous studies on anisotropy strength and tip radius [43,53].

### 3.4 Calculation time

The time required for each calculation when varying the random terms and distributions is measured and listed in Table 3. The calculation time is 7000 s when using random numbers following a uniform distribution, whereas it is more than twice that when using random numbers following the Gaussian distribution. This result indicates that the computational cost of generating random numbers following the Gaussian distribution makes up a significant portion of the total computational cost. The Box–Muller transformation is used to generate random numbers following the Gaussian distribution in this study. However, this transformation requires computationally expensive functions such as logarithmic functions, trigonometric functions, and root signs, which require two random numbers following a uniform distribution.

**Table 3:** Comparison of calculation time for each distribution and random terms in 100,000 time step.

random terms	distribution	calculation time
χ	Uniform distribution	7210 s
χ	Gaussian distribution	15634 s
∇·q	Uniform distribution	7842 s
∇·q	Gaussian distribution	18852 s

## 4 Conclusion

In this study, the effect of random terms in the phase-field method on the dendrite shape was investigated by changing the introduced term, distribution, and variance of a random number. By introducing the random terms to the equations of the difference between the order parameter and temperature, we found that the difference in the magnitude of the noise increased by a factor of 10^8^. This discrepancy arises due to the lack of normalization for each variable, suggesting the necessity of appropriately adjusting the magnitude of the noise according to the introduced terms. As for the distribution, there was no clear difference in the shape of the dendrites between the uniform distribution and the Gaussian distribution. This result suggested that similar outcomes can be obtained from Phase-field analyses based on both uniform and Gaussian distributions, providing valuable insights. The magnitude of the noise was set empirically in a previous study by Kobayashi [25]. This measurement clarified the trend of the dendrite shape as the parameters of a random number were changed and achieved a random number design that was close to the experimental value. This result provided a meaningful contribution, as it allows the magnitude of the noise in Phase-field simulations, which has traditionally been set empirically, to be determined through quantitative analysis. Finally, from the viewpoint of computational cost, we conclude that the use of random numbers following a uniform distribution for χ is better. In particular, the computational cost varied by approximately a factor of two depending on the choice of probability distribution. We believe our research facilitates the development of an efficient approach to accelerate Phase-field simulations.
